# A case report of splenic rupture related to colonoscopy

**DOI:** 10.1093/jscr/rjaf313

**Published:** 2025-05-17

**Authors:** Navid Moghimi, Markus A Puchner

**Affiliations:** Department of Gastrointestinal & General Surgery, Hvidovre Hospital, 2650 Hvidovre, Denmark; Department of Gastrointestinal & General Surgery, Hvidovre Hospital, 2650 Hvidovre, Denmark

**Keywords:** splenectomy, splenic rupture, colonoscopy

## Abstract

Splenic rupture is a rare but serious iatrogenic complication of colonoscopy, potentially leading to hemodynamic instability. When common complications such as post-polypectomy syndrome and perforation are excluded, physicians must maintain a high level of suspicion for splenic injury, particularly in patients presenting with abdominal pain after the procedure. This report describes an emergency splenectomy performed on an 80-year-old male following a routine colonoscopy. Seven hours of post-procedure, the patient presented abdominal pain, syncope, sweating, and hypotension. Computed tomography imaging revealed a ruptured spleen with a 7-cm parenchymal hematoma, a significant blood halo surrounding the liver, and a large blood accumulation in the pelvis. The patient underwent an emergency splenectomy and was discharged 6 days later without complications.

## Introduction

Colonoscopy is a widely employed procedure for colorectal cancer screening and diagnosis. While it is generally considered safe, complications such as bowel perforation and intraluminal bleeding, though rare, may occur. Splenic injury is an even less common but serious complication of colonoscopy, with a reported mortality rate ranging from 5% to 10% [1–3]. Although the mechanisms behind splenic injury are not fully understood, factors like traction on the splenocolic ligament, direct pressure from the endoscope at the splenic flexure, and external abdominal pressure during insertion have been suggested as contributing causes [4]. Management strategies vary, with laparotomy, selective arterial embolization, and conservative approaches being considered depending on the clinical scenario. In hemodynamically unstable patients, an emergency splenectomy is often required. Here, we present a case of splenic injury following colonoscopy, successfully managed with surgical intervention.

## Case report

An 80-year-old male underwent a routine annual colonoscopy due to his history of chronic constipation. The patient was receiving anticoagulant therapy with rivaroxaban, which had been discontinued 3 days before the colonoscopy.

The bowel preparation for the procedure was adequate, although the colonoscopy was technically challenging due to loop formation, resulting in a procedure duration of ~60 min. Despite these difficulties, the colonoscopy was completed successfully, and the patient was discharged in stable condition, reporting no pain.

**Figure 1:**
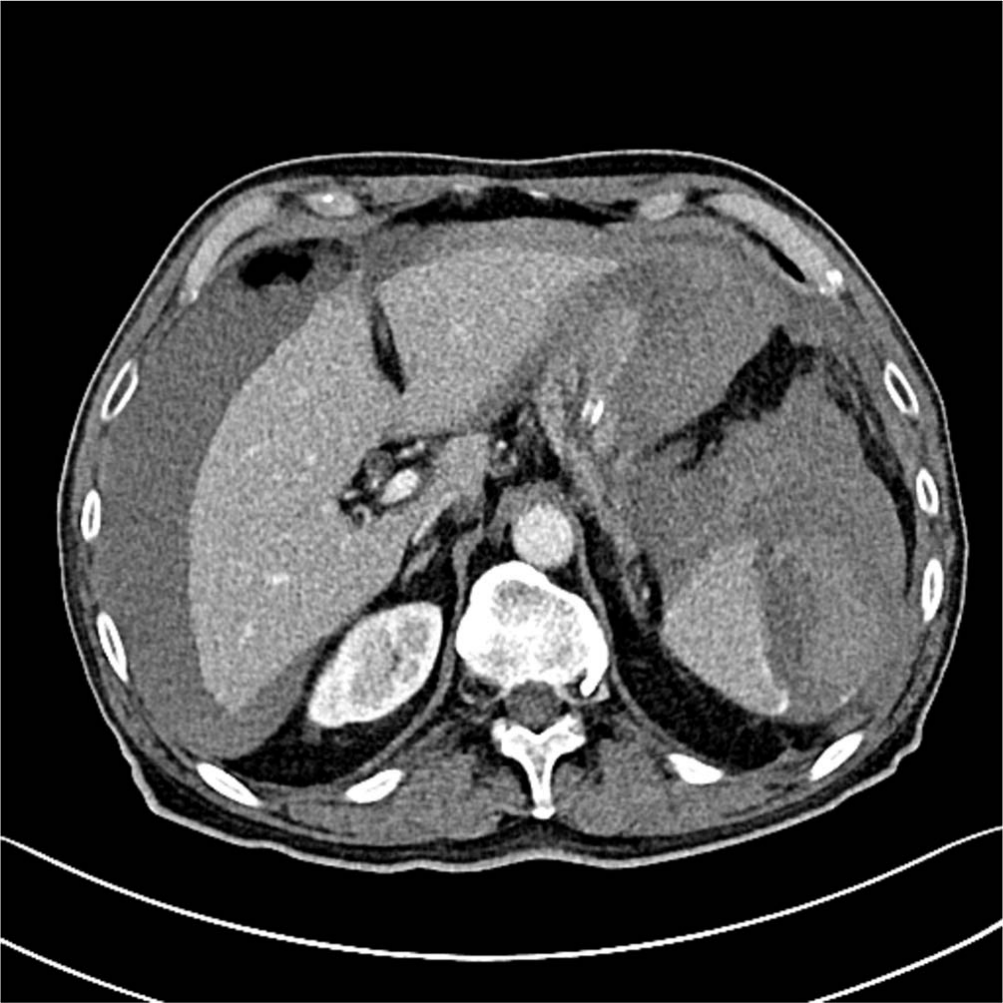
The CT scan reveals splenic hematoma with a significant blood halo surrounding the liver.

**Figure 2:**
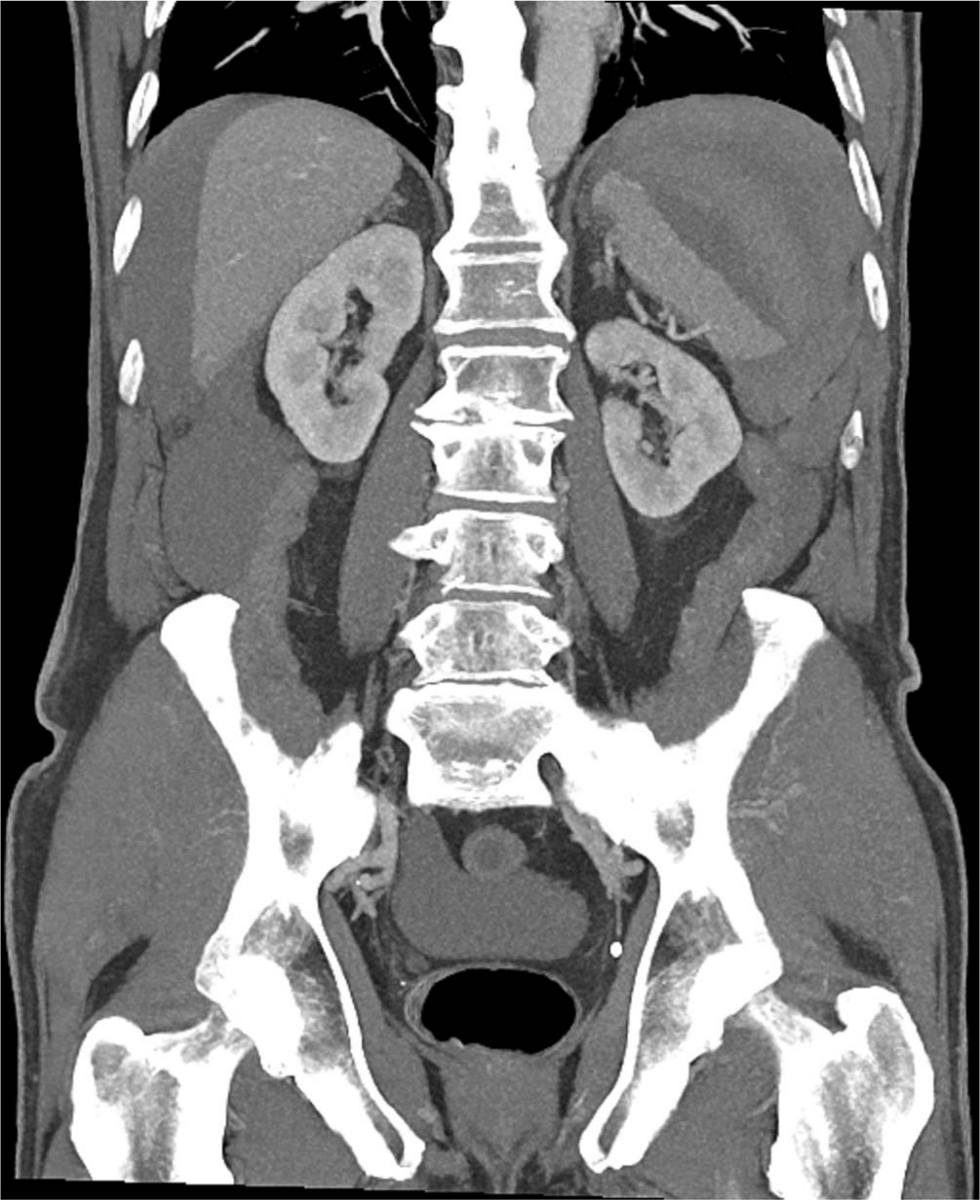
Grade III splenic injury with intraparenchymal hematoma larger than 5 cm [5].

Seven hours post-procedure, the patient presented to the emergency department with abdominal pain, syncope, diaphoresis, and bloating. His blood pressure was 80/50 mmHg, and his heart rate was 120 beats per minute. Abdominal examination revealed tenderness in the left hypochondrium, and his hemoglobin level was 6.2 g/dl with a hematocrit of 38%. A contrast-enhanced abdominal computed tomography (CT) scan showed a ruptured spleen with a 7-cm parenchymal hematoma, a significant blood halo surrounding the liver, and a large blood accumulation of 15 × 12 cm in the pelvis (Figs 1 and 2).

Initial management included intravenous fluid resuscitation and the transfusion of three units of packed red blood cells. Given the patient's deteriorating clinical condition and the significant amount of blood within the abdomen, surgical intervention was necessary.

Surgical exploration via median laparotomy revealed a significant hemoperitoneum. The spleen was mobilized, and an actively bleeding lesion near the splenic flexure, adherent to the left colic angle, was identified. A hemostatic splenectomy was performed with full hemostasis achieved.

The patient's postoperative recovery was uneventful, and he was discharged home on postoperative Day 6 with appropriate antibiotics and vaccinations.

## Discussion

Splenic injury is rare, with over 100 reported cases and an estimated incidence ranging from 0.002% to 0.033%. This is lower than the incidence of other known complications, such as bowel perforation (0.035%–0.073%) and intraluminal bleeding (0.065%–0.231%) [3, 6]. The mortality rate from splenic injury following colonoscopy ranges between 5% and 10% [2, 3].

The typical presenting symptoms of splenic injury include abdominal pain (46.1%), left shoulder pain (40.91%), dizziness or lightheadedness (7.79%), and syncope (5.19%) [7]. In this case, the patient experienced localized abdominal pain in the left upper quadrant, accompanied by tachycardia (120 bpm) and hypotension (80/50). His hemoglobin level was found to be 6.2 g/dl, which further supported the diagnosis of splenic rupture, confirmed by CT imaging.

When abdominal pain presents after a colonoscopy, differential diagnoses should include bowel perforation, post-polypectomy syndrome, and splenic rupture. In this case, perforation was ruled out due to the absence of free air on imaging, and post-polypectomy syndrome was excluded because the colon appeared normal on CT.

The precise mechanism of splenic rupture following colonoscopy remains unclear. One theory suggests that excessive traction on the splenocolic ligament during colon navigation may cause detachment of the splenic capsule from the parenchyma, resulting in laceration [3, 8]. Additionally, anatomical variations in the splenocolic ligament, such as a shorter ligament, may predispose patients to splenic injury. Moreover, excessive external pressure on the left upper quadrant during the procedure may contribute to blunt trauma to the spleen [3, 9]. Patients with prior abdominal surgeries or intra-abdominal infections, which increase adhesions and restrict splenic mobility, are also at greater risk for splenic rupture [10].

In this case, the injury likely resulted from a combination of external abdominal pressure and endoscopic traction. The looping observed in the sigmoid and transverse colon required significant angulation and traction at the splenic flexure, potentially increasing the risk of injury to the spleen via the splenocolic ligament.

Splenic rupture can be managed non-operatively or operatively, depending on the patient's hemodynamic stability and the extent of the rupture. Non-operative management involves monitoring in an intensive care unit and transfusion of blood products as necessary. However, failure rates for non-operative management can be as high as 10% [11]. In cases of hemodynamic instability or significant splenic injury, splenectomy remains the definitive treatment [4, 12].

## Conclusion

Splenic rupture, though rare, is a potentially life-threatening complication of colonoscopy. The injury typically occurs due to excessive traction on the splenocolic ligament, leading to avulsion or tearing of the splenic capsule. When common post-procedural complications like perforation and post-polypectomy syndrome have been excluded, clinicians must maintain a high index of suspicion for splenic rupture in patients presenting with abdominal pain following colonoscopy. Management depends on the patient's hemodynamic status, with splenectomy often required in unstable patients.
